# A novel multi-alignment pipeline for high-throughput sequencing data

**DOI:** 10.1093/database/bau057

**Published:** 2014-06-19

**Authors:** Shunping Huang, James Holt, Chia-Yu Kao, Leonard McMillan, Wei Wang

**Affiliations:** ^1^Department of Computer Science, University of North Carolina, Chapel Hill, NC 27599, ^2^Department of Computer Science, University of California, Los Angeles, CA 90095, USA

## Abstract

Mapping reads to a reference sequence is a common step when analyzing allele effects in high-throughput sequencing data. The choice of reference is critical because its effect on quantitative sequence analysis is non-negligible. Recent studies suggest aligning to a single standard reference sequence, as is common practice, can lead to an underlying bias depending on the genetic distances of the target sequences from the reference. To avoid this bias, researchers have resorted to using modified reference sequences. Even with this improvement, various limitations and problems remain unsolved, which include reduced mapping ratios, shifts in read mappings and the selection of which variants to include to remove biases. To address these issues, we propose a novel and generic multi-alignment pipeline. Our pipeline integrates the genomic variations from known or suspected founders into separate reference sequences and performs alignments to each one. By mapping reads to multiple reference sequences and merging them afterward, we are able to rescue more reads and diminish the bias caused by using a single common reference. Moreover, the genomic origin of each read is determined and annotated during the merging process, providing a better source of information to assess differential expression than simple allele queries at known variant positions. Using RNA-seq of a diallel cross, we compare our pipeline with the single-reference pipeline and demonstrate our advantages of more aligned reads and a higher percentage of reads with assigned origins.

**Database URL:** http://csbio.unc.edu/CCstatus/index.py?run=Pseudo

## Introduction

Many next-generation sequencing analyses rely on an alignment to a reference genome. Generally, this alignment is to a single reference sequence, regardless of the underlying organism’s ploidy. When parental haplotypes differ in their similarity to the reference sequence, a significant alignment bias can result (1, 2). This is particularly problematic when allele counts are treated as quantitative measures. Therefore, it is important to use proper reference sequences for read mapping, as it will inevitably affect the accuracy of alignments in terms of mapping ratio (i.e. the ratio of reads that are mapped) and mapping locations.

The most commonly used approach is to map reads to a standard reference and rely on the error tolerance of the aligner to compensate for the genomic variations of the target sequence. Gregg *et al.* (3) aligned F1 hybrids from reciprocal crosses between the isogenic mouse strains CAST/EiJ and C57BL/6J to the NCBI37/mm9 mouse genome and transcriptome to study parent-of-origin effects. However, this approach favors reads with reference alleles, and it is worth noting that the mouse reference genome is largely based on C57BL/6J. This results in a systematic bias, called the reference bias. To reduce this reference bias, Degner *et al.* (2) proposed masking every known polymorphic location in the reference genome with a third allele. This approach reduces the total number of reads aligned because the added masked alleles always introduce mismatches, which all aligners attempt to minimize. In fact, unmapped reads result when the best mapping considered has excessive mismatches. In RNA-seq experiments, this reduction in mapped reads leads to underestimation of expression level of genes with variations (4). Several attempts have been made to create a sample-specific reference genome or transcriptome for alignments. Keane *et al.* (5) aligned reads from F1 cross of C57BL/6J × DBA/2J to a C57BL/6J-based reference genome and an approximate DBA/2J genome where known DBA/2J variants, primarily single nucleotide polymorphisms (SNPs), were substituted into the reference. Turro *et al.* (4) proposed a hybrid pipeline that first aligned reads to a reference genome to call SNPs, and then realigned the same reads to a customized transcriptome with the discovered SNPs incorporated. As single-base substitutions do not change genome coordinates, it is straightforward to embed SNPs. However, this method cannot be easily generalized to other frame-shifting variants such as small indels, inversions and copy number variants to which a sequence aligner is more sensitive. Rozowsky *et al.* (6) proposed AlleleSeq for constructing a modified diploid genome by inserting SNPs and indels into the reference genome and using this diploid genome as the reference during alignment to avoid errors caused by reference bias. Although AlleleSeq is similar to our proposed pipeline, it is limited to diploid organisms. Moreover, as shown below, it analyzes differential expression at variant positions, which will become more difficult as the density of variants increases.

After reads are mapped, the relative read counts in specific regions of the sequence are often used to quantify abundance within a genomic region. In DNA sequencing (DNA-seq), local read counts are used to estimate copy-number gain or loss (7, 8). In RNA sequencing (RNA-seq), local read counts are used to quantify gene expression levels and to identify the isoforms expressed (4, 9). In diploid organisms, researchers have been interested in assessing the differential expression levels between parental haplotypes (i.e. parent-of-origin or allele effects). In a typical analysis of differential expression, the read coverage at each known variant position are partitioned according to allele and then used to estimate the imbalance (3, 5, 6). Statistical corrections to the read counts are required when the density of local variations allows multiple variants to fall in the same read or read-pair. Thus, in regions with dense genomic variations, the quantitative use of read counts is complicated both by an inability to align, and a difficulty of establishing the independence of each variant observation.

We propose a new read annotation pipeline that overcomes most of these problems. It uses multiple alignments and a merging process in an attempt to resolve a given read’s origin.

First, we leverage the existing databases of genomic variants to build custom reference genome sequences for all parental haplotypes, each of which is used in an independent alignment procedure. We call these synthetic genomes **pseudogenomes**. Unlike most previous methods, different types of variations, such as SNPs, indels and structure variants (SVs), can all be integrated into the pseudogenomes. As the coordinates in the alignment to pseudogenomes are no longer relative to the reference because of the incorporated indels and SVs, we remap all positions back to the reference coordinate system after alignment. This remapping enables comparisons of the pseudogenome alignments and allows us to use existing annotations (i.e. positions of gene exons and functional elements), which are generally based on the reference sequence’s coordinates.

In a second stage, we merge alignments to multiple pseudogenomes and assign an origin to every read. Because of the previous multi-alignment process, each read may be aligned to more than one pseudogenome. Even within the same pseudogenome, depending on the settings of an aligner, reads can be mapped to multiple locations. Although discarding reads with multiple mappings is a common practice (1, 5, 10, 11), their exclusion can lead to additional biases in downstream estimation (4). In the merging stage of our pipeline, we resolve such multiple mappings where possible by keeping the best choice based on several well-defined criteria. Meanwhile, each read is labeled with its most likely origin based on comparing its mappings in multiple pseudogenomes. This label facilitates downstream analyses, as read counts instead of allele counts are used to assess differential expression, which requires less severe independence assumptions (measures based on the origin labels of a read need only consider the likelihood that reads are from the same transcript, whereas measures based on allele counts at a particular genomic position must, in addition, consider the likelihood that nearby variants are from the same read).

## Methods

In this section, we describe our pipeline for annotating multi-parental sequencing data. For the purpose of discussion, we will assume that the data set being analyzed is RNA-seq from a two-founder diallel cross. The diallel produces samples from crossing two isogenic parental genomes. Our pipeline is not limited to analyzing diallels, nor is it limited to RNA-seq analysis, as discussed in the ‘Discussion and conclusion’ section. For comparison purposes, we also consider a second analysis pipeline that uses a single reference genome and attempts to achieve similar annotations. In all fairness, this single-reference pipeline is only an approximation to the front ends of other published methods. We have deliberately attempted to separate the annotation phase of sequence analysis from subsequent analyses in our pipeline. Our new pipeline consists of multiple alignments that incorporate all known genetic variants into a genomic model followed by annotation and merging. Assessments of the differential expression levels owing to parent, allele or slice variants are considered downstream uses of our annotations. We contrast our multi-alignment-based approach with a representative reference-based pipeline and highlight their major differences.

### Single reference pipeline

In traditional reference-based alignment pipelines, short reads from high-throughput sequencers are first mapped, and genetic variation is considered afterward. There are significant advantages in using a standard reference genome. In addition to supplying a standard coordinate system for comparison between target genomes, reference coordinates anchor nearly all of the genome’s functional annotations, such as gene/exon locations, transcription factor binding sites and notations of common variants. When all samples are aligned to this reference, genomic comparisons are significantly simplified. However, the mappability to the reference genome is reduced if a sample has a large number of variations from the reference. This results in either a reduction in the number of reads mapped and/or an increase in mapping errors. If the number of errors exceeds the aligner’s tolerance, the read will be simply dropped from the output and its information will be lost. In short, a sample that is genetically distant from the reference will typically align fewer reads and with reduced confidence than a sample that is closer to the reference.

There is an extra step of annotating the reads after they are mapped in the alignment. Specifically, the known allelic variations between the parental genomes are used to assign its origin. Consider a diallel cross of two inbred mouse strains as an example. If a mapping shares three SNPs with the maternal strain but only one with the paternal one, the single-reference pipeline assumes that the mapping is from the maternal side. If such counts are the same, suggesting an equal chance of coming from either one, then the strain origin of this mapping cannot be determined.

### Multi-alignment pipeline

One of the flaws with the single-reference pipeline, applied to an entirely homozygous inbred sample, is the fact that the alignment process does not take into account known sequence differences between the given inbred and the reference. Instead, this is typically handled later during analysis or as a post-alignment annotation (as described in the ‘single-reference pipeline’ section). One of the major differences in our new pipeline is to take advantage of known allelic differences as early in the pipeline as possible.

#### MOD format

We first propose a general-purpose framework for mapping back and forth between genomes, which is suitable for both short reads and genomic annotations. It is facilitated by a mapping file, called a MOD file, that describes all variations between a reference and a target genomic sequence (12). MOD files provide a generative mapping from reference sequence to a target sequence that incorporates all known structural variants (SNPs, indels, translocations and inversions).

The MOD format is composed of instructions that transform one genome sequence into another. It is essentially an edit transcript relating two strings (13), and it provides a basis for quantifying the similarity of two sequences. A MOD file is not necessarily unique, nor do we make any claims with regard to minimality. We call the genome before transformation **the source** and the one after **the destination**. Each MOD file is directional, i.e. always from the source to the destination.

**Figure 1.**
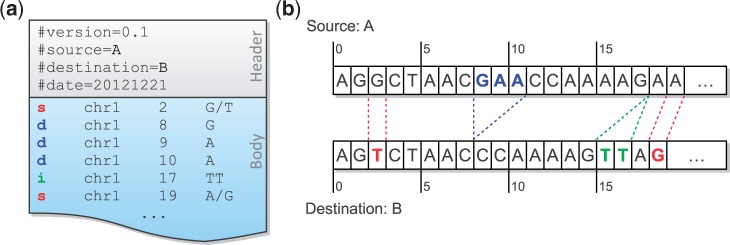
A MOD file example (**a**) and the corresponding sequences of the source and the destination (**b**). There are two SNPs between these sequences, and they are represented as two s-instructions at source positions 2 and 19. A three-base deletion (from source positions 8–10) is observed, and it is broken up into three d-instructions. The insertion after position 17 is directly added to the MOD file without any conversion because of its atomicity.

A MOD file consists of two parts (Figure 1a): a header and a body. The header includes the metadata of the transformation, such as the version of the MOD format, the source, the destination and so forth. The body holds the instructions, each of which has its affected position and arguments. Positions are all stored in the source coordinate system, and the bases before and after modification are included in the arguments.

There are three basic types of instructions defined in the MOD format: **s-**, **d-** and **i-instructions**. They describe single-base substitutions, single-base deletions and insertions, respectively. All instructions are **atomic**, in that they reference no more than one position from the source. It is obvious that both s-instructions and d-instructions are atomic. For i-instructions, we merely add new sequence after an anchor position in the source without altering any base; thus they are also atomic.

One way to generate a MOD file is to convert common variant calls into instructions. For example, SNPs and genomic insertions can be directly changed to s-instructions and i-instructions, respectively. For genomic deletions, we need to break each of them up into single-base deletions before converting to d-instructions (Figure 1a and b). Notice that the position information in adjacent d-instructions is redundant. However, the design choice of keeping all instructions atomic facilitates later MOD-file manipulations, whose advantages are considered to outweigh this slight redundancy. Moreover, the additional space overhead is recovered when MOD files are compressed.

Complex structure variants, such as tandem duplications, inversions and translocations, can be described by the current set of instructions. For example, a tandem duplication is represented by repeating an i-instruction at the same location, whereas inversions (or translocations) are implemented by a series of d-instructions at the source sequence position and a corresponding i-instruction of the inverted (or transferred) sequence at its new position. We recommend annotating such coupled sets of instructions using comments following the instructions.

#### Pseudogenome construction

MOD formatted files provide a generative procedure for transforming a source sequence to a destination. We can generate a pseudogenome for the inbred based on the reference and known genetic variants (SNPs, insertions and deletions).

In our pipeline, the *MOD Interpreter* will execute the instructions of a MOD file for an inbred strain and incorporate variants into the reference genome sequence to obtain a pseudogenome. The detailed procedure is shown in Algorithm 1. The generated pseudogenome is then used in the alignment process instead of the reference genome, so the end result of alignment is a BAM file (14) using the coordinate system of the pseudogenome.

**Algorithm 1 Pseudogenome Construction**

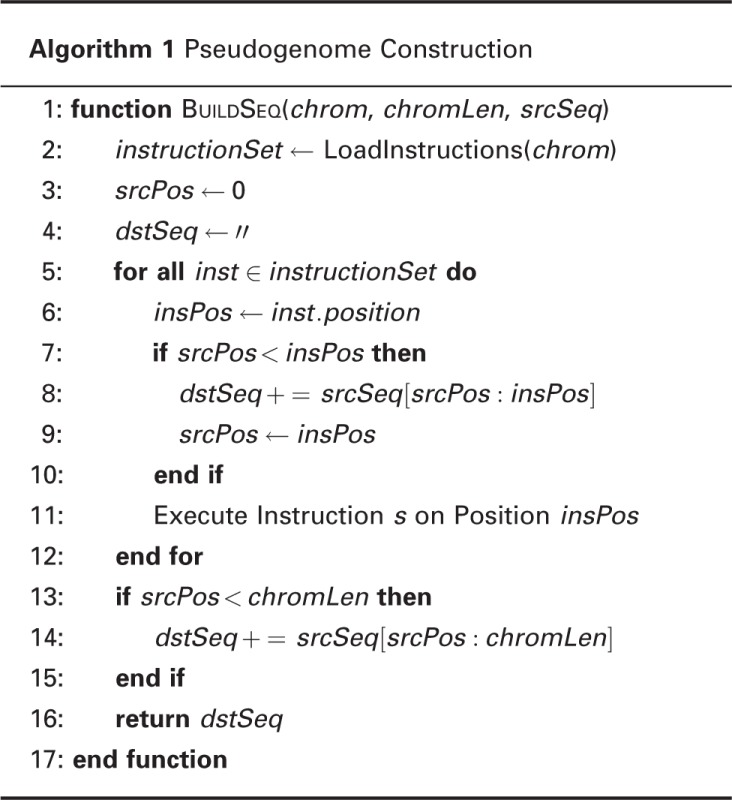

#### Pseudogenome alignment and annotation

Separate alignments create problems when comparing samples. If we tried to compare a CAST/EiJ inbred to a PWK/PhJ inbred using only the pseudogenome alignments, there would be two different genomic coordinate systems in play. To alleviate this issue, we use the same set of known allelic differences incorporated into the pseudogenome to translate all of the mapped reads back to the reference coordinate system. This involves going through each mapped read and adjusting the mapping position, cigar string and edit distance to match the reference genome instead of the pseudogenome. It is done by scanning a MOD file and accumulating the number of shifted bases affected by d-instructions and i-instructions. For every pair of corresponding regions in the two genomes, we record a pair of offsets. Given a position in the source, we first look up in the source offsets to find out in which region it falls and then compute its destination position. This step is performed by a Python program called Lapels in our pipeline (Algorithm 2).

Finally, each mapping is annotated with a series of tags to preserve information from the original pseudogenome alignment. To assess the mapping quality, each remapped read retains the cigar string and edit distance from the original pseudogenome mapping as tags. These tags allow us to calculate the original quality scores and preserve information regarding the differences between the reference and pseudogenome mappings for that read.

**Figure 2.**
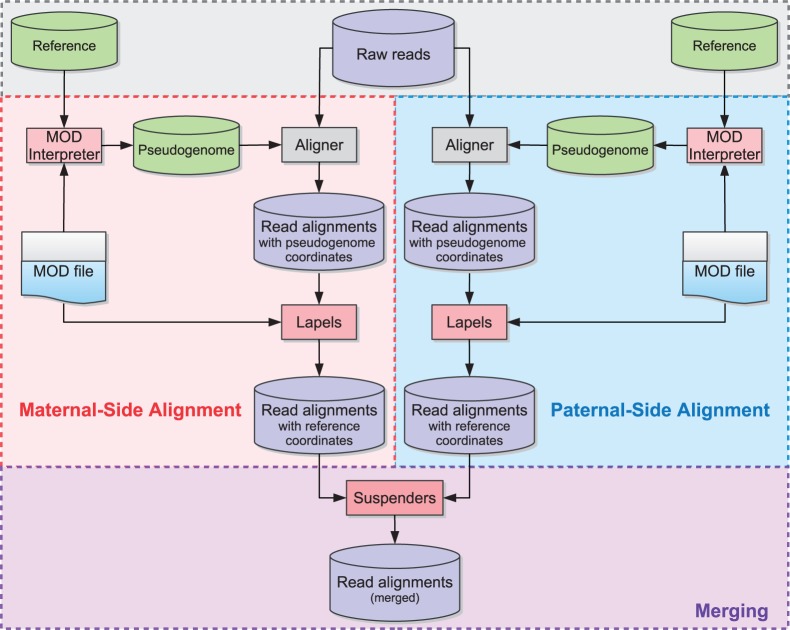
Multi-Alignment Pipeline for a diallel cross. Here we assume that the organisms being considered are diploid. The first step is to create two pseudogenomes using the list of known allelic differences, align the same reads to both pseudogenomes and convert the mappings back to the reference coordinate system. Next we merge the two alignments and keep only the best mappings to either pseudogenome in the final merged file.

Unfortunately, alignments are more difficult to assess for multi-parental crosses and diploid organisms in general because there are multiple sets of allelic differences to influence the alignment. In a diallel sample, there are two sets of allelic differences to take into account. We address this problem by constructing pseudogenomes for all contributing founder genomes, performing separate alignments of the full data set to each pseudogenome, and remapping them back to a reference genome while annotating differences as described earlier (Figure 2).

#### Merging—comparing alignments

Next, we consider all annotated alignments as input and merge them into a single output by choosing the best mapping for each read. This is performed by Suspenders in our pipeline (15). Suspenders sorts the BAM files by read name and systematically compares each alternative mapping. For each read, it extracts all mappings from each Lapels-annotated BAM file before comparing the results. As explained earlier, information from the original pseudogenome alignment is preserved by the annotation step.

The first step in the merge process is to identify the origin of each mapping. To do this, Suspenders first identifies **identical mappings** based on the mapping start position in the reference genome (chromosome and coordinate), its cigar string in the reference genome, its pairing (either paired or unpaired) flag, its fragment end flag (either first or second) and its quality score [e.g. the Bowtie end-to-end score (16)] from the pseudogenome mappings. The start position and cigar string assure that the two mappings in question cover the same genomic interval in the reference coordinate system. If the pairing and fragment end flags are also the same, it indicates that the read fragment was mapped in the same way in the separate pseudogenome alignments. The final criterion of comparing quality scores implicitly takes allelic differences into account and is discussed in greater detail later. If a read’s mappings to two or more pseudogenomes are identical, Suspenders merges the mappings into one logical unit and tags the mapping with a bit vector to identify the origin. For example, for a diallel sample, it would tag each mapping with a 2-bit flag set indicating its origin (01: first parent, 10: second parent, 11: either parent). Read mappings that uniquely map to a single pseudogenome are tagged according to their source (i.e. by setting a single bit).

**Algorithm 2 Remapping Reads**

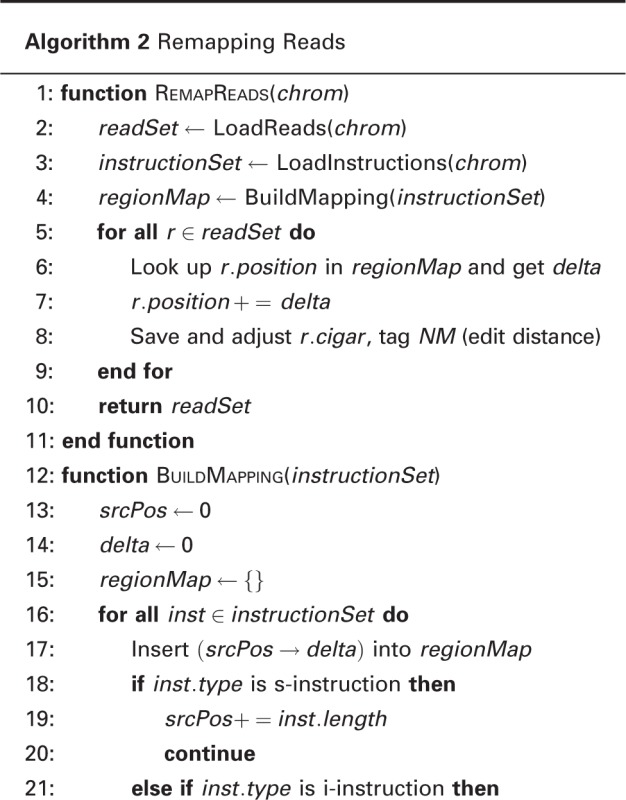

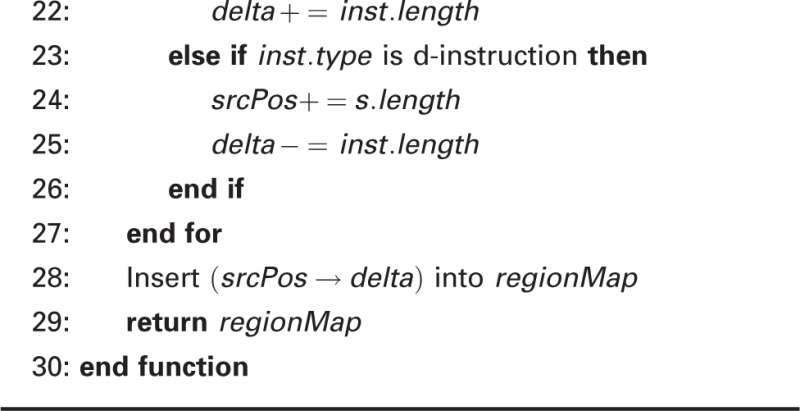

For paired-end reads of a fragment, we ‘link’ parental origins. The main idea is that if one read from a fragment can be assigned to a parent unambiguously, we infer that its mate also came from that same parent, even if the mate contains no informative variants. To illustrate the significance of linking, in our example single-reference pipeline (‘Single-reference pipeline’ section), each read is treated independently during the annotation step even if it is mapped as a paired-end read, thus requiring the presence of an informative allele in the read to assign its origin. This approximates the common process of examining alignments only at informative variant positions, while retaining the ability to detect whether two variants fall on the same read. In our new pipeline, if two read mappings are properly paired, then they are treated as a single unit throughout the entire merging process. This means that (i) the mappings of two properly paired reads will be marked with the same parental origin, (ii) the quality score will be calculated once based on the mismatches and indels from the paired mapping and (iii) the merge always prefers a paired-end mapping to one or two single-end mappings from the pair.

#### Merging—filter pipeline

Entering into the filtering section of the pipeline, we have a set of possible mappings for a given fragment where each mapping is marked with an origin flag. By sending the set through a series of filters, we remove mappings from the set until only one possible mapping remains for each fragment. Before filtering, Suspenders checks to see whether any of the possible mappings are mated paired ends. If so, it immediately removes all unpaired mappings from consideration, as we prefer a paired mapping over an unpaired one. If there are no paired-end mappings, the mappings are grouped depending on whether they are the first or second read from the fragment, and a mapping set from each read is independently sent through the Suspenders filters. The two unpaired ends of the same fragment may be filtered in different ways because they are handled separately.

**Figure 3.**
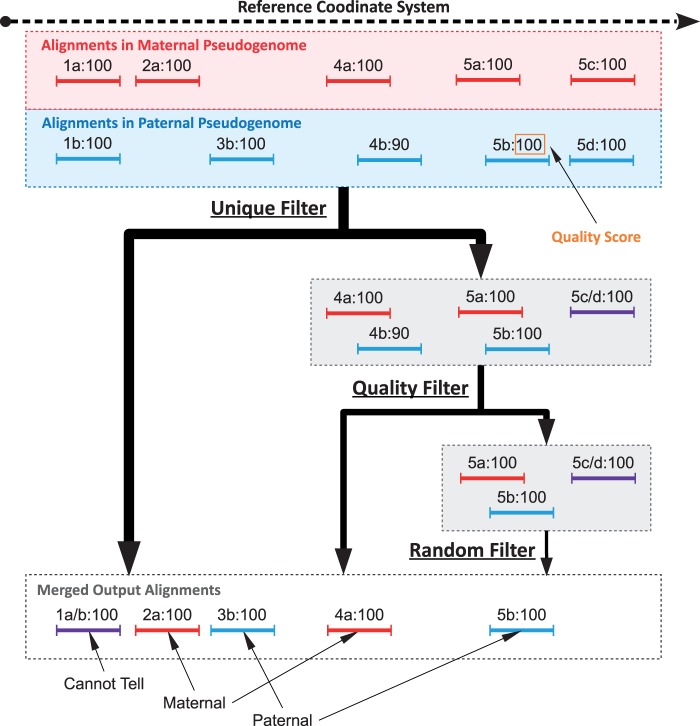
Sample filter path for mapping sets of five reads in a diallel cross labeled such that ‘4b:90’ is mapping ‘b’ of read 4 with a score of 90. Before filtering, 1a and 1b are combined into 1a/b because they have the same position and score in both mappings. Additionally, 5c and 5d are also combined. The mapping sets of reads 1, 2 and 3 are all outputted by the Unique filter, as there is a single positional mapping for each. The mapping sets of reads 4 and 5 have multiple mappings, so they are diverted to the Quality filter by the Unique filter. The Quality filter outputs 4a, as only one mapping of read 4 has the best score (100 compared with 90). The mapping set of read 5 has three mappings with identical scores and is therefore diverted to the Random filter, which chooses one mapping arbitrarily. As there are three in the set, each one has a 33.3% chance of being chosen, with 5b being the arbitrary choice in this example.

The next step is to send the mapping sets through a series of three filters (shown in Figure 3): Unique, Quality and Random. If a mapping is output by a filter, we add additional annotation to indicate the chosen mapping as coming from that filter. The Unique filter identifies the reads whose mapping sets contain a single mapping and outputs these mappings. These include all reads having a unique mapping to only a single pseudogenome, and reads mapping to multiple pseudogenomes with single identical mapping as defined previously. In short, the Unique filter outputs simple cases where a read (or two paired reads) has one unique mapping across all pseudogenomes. In Figure 3, reads 1, 2 and 3 each have a single unique positional and score mapping, so they are all output by the Unique filter.

As mentioned earlier, the score comparison is where this pipeline implicitly takes into account allelic variations in the sample. An aligner typically uses a quality score to quantify the mapping quality, which is a function of the number of mismatches, insertions and deletions. Only the mapping(s) with the best score are outputted. For example, TopHat uses the Bowtie scoring scheme (16) when reporting possible mappings (17–19). Assume that a read aligns to multiple pseudogenomes that straddle an informative variant caused by a SNP. The mappings to pseudogenomes with the matching variant will have fewer mismatches than that to genomes with the alternate allele. As sequencing errors are attributes of the read, they contribute mismatches equally to all pseudogenome mappings. In places with no informative alleles, an aligner will report mappings to all genomes with identical number of mismatches. Additionally, if there are multiple variants under a read’s mapping, the read may be mapped to multiple positions in the genome, but usually only the best mappings are reported. The Quality filter attempts to simulate this behavior by keeping only the best mappings and their corresponding references. Before the filters, identical mappings (with same coordinates and scores) were combined into a single unit. However, the mappings were not combined if their scores were different. The Unique filter treats them as two different mappings from distinct origins and passes them to the Quality filter.

Read fragments with multiple mappings (possibly to the same position) are passed to the Quality filter. For each read fragment, we regenerate the original scores of the pseudogenome mappings from the stored cigar strings and edit distances saved during the annotation phase when remapping back to the reference. If only one mapping has the best score, then that mapping is outputted by the Quality filter. In Figure 3, the mapping set of read 4 has two different mappings (one from maternal and one from paternal). The maternal mapping has a higher quality score, so it is outputted by the Quality filter.

However, if multiple mappings of a mapping set have the same best score, then we pass those mappings to the Random filter as a last resort. The Random filter will choose only one from the set at random to keep in the merged result. Each chosen mapping is tagged with the filter it came from, so the option to remove all mappings from the Random filter can be performed in downstream analysis. In Figure 3, the mapping set of read 5 has three possible mappings with identical scores. Each mapping has a 33.3% chance of being chosen for the final output. After each read fragment has been processed using the filter pipeline, the final result is a single merged file containing at most one mapping for each single-end read and at most one paired mapping or two unpaired mappings for each paired-end read. Additionally, each read mapping is tagged to identify its pseudogenome origin and the filter that output it during the merge process.

## Results

We evaluated our pipeline using a full diallel cross of two wild-derived inbred mouse strains: CAST/EiJ and PWK/PhJ. We use the notation of CAST × PWK to represent the cross whose maternal and paternal parents are CAST/EiJ and PWK/PhJ, respectively. Likewise, the reciprocal cross is denoted by PWK × CAST.

We first extracted mRNA from brain tissues of 10 female samples (five for each cross). Then we used Illumina HiSeq 2000 platform to sequence the transcribed cDNA and obtained around 1.2G paired-end reads with 100 bp (2 × 100). The number of reads per sample is shown in Table 1.

**Table 1.** Total number of reads for 10 samples in two F1 hybrid crosses

Strain	CAST × PWK	PWK × CAST
Sample 1	115 936 064	119 926 340
Sample 2	87 988 306	90 706 788
Sample 3	142 479 432	170 423 066
Sample 4	137 698 560	92 829 168
Sample 5	137 953 398	93 801 072
Total	622 055 760	567 686 434

To generate MOD files for the two founder strains, we first extracted SNPs and indels from the VCF (20) files (downloaded from ftp://ftp-mouse.sanger.ac.uk/REL-1105/). Only high-confidence SNPs and indels for the 19 autosomes and X were incorporated into the MOD files. Variants on mitochondria (M) were extracted from other sources (http://cgd.jax.org/datasets/popgen/diversityarray/yang2009.shtml). The MOD files used in this article can be found at http://www.csbio.unc.edu/CCstatus/index.py?run=Pseudo. For each MOD file, the statistics for the whole genome are summarized in Table 2.

**Table 2.** Statistics of MOD files for CAST/EiJ and PWK/PhJ

Strain	s-instructions	d-instructions	i-instructions
CAST	17 674 364	4 834 899	4 206 776
PWK	17 202 935	4 715 249	3 457 436

The counts are in units of base pairs. For s-instructions and d-instructions, they are just the numbers of instructions, respectively. For i-instructions, the counts are derived from adding up the number of bases in each inserted sequence.

We ran several tools with their default parameter settings in both pipelines. RNA-seq reads were aligned against the genomes by Tophat (v2.0.5). In the multi-alignment pipeline, we used Lapels (v1.0.4) to map each read coordinate back to the reference, and Suspenders (v0.2) to merge and tag mappings. Only one mapping per read was reported in the final output.

### Comparison of mapping ratio

**Figure 4.**
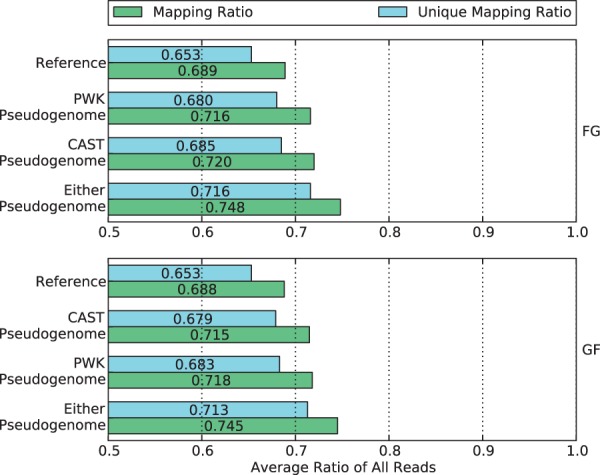
Mapping ratio and unique mapping ratio of reads to the reference genome, two pseudogenomes and either pseudogenome. Using a single pseudogenome provides a gain of ∼3% over the reference genome, and using both almost doubles the gain to 6%.

We examined the fraction of mapped reads from alignments to pseudogenomes, and compared it with the fraction of the same reads when mapped to the standard reference genome. This is an imperfect comparison, as we consider only whether a read maps without considering the accuracy or quality of the mapping. The mapping ratios are shown in Figure 4.

Observe that more reads are mapped to each parental pseudogenome than to the reference. The percentage gain is ∼3% for both pseudogenomes in the two crosses. A similar increase can be seen in the percentages of uniquely mapped reads. This suggests that by integrating the variations of parental strains into the reference, we have obtained two better genomes for the reads to align to.

If we consider whether reads mapped to either or both pseudogenomes, the combined recovery rate gain almost doubles to around 6%. To take advantage of this gain, we use the merge process of our method to combine the two sets of alignments in the following section.

### Comparison of parental origin labeling

After reads are mapped, our new pipeline and single-reference pipeline next attempt to label the pseudogenome origin of every read where possible. This is a crucial step for downstream analyses, which leverage the labels to determine differential gene expression between the parental strains.

As common aligners can allow a small amount of mismatches during alignment, reads containing SNPs may still be mapped to the reference. After alignment, the single-reference pipeline can check every SNPs positions in the reads and count the numbers of maternal alleles and paternal alleles. Then the ratio of the two allele counts is used to determine the parental origin of each reads. If no maternal and paternal alleles are observed or both counts are the same, it will be classified into the ‘can’t tell’ category. Otherwise, we choose to label the mapping according to which the allele count is greater.

In our proposed pipeline, the label for each mapping is determined during the merging stage after considering the mappings to multiple pseudogenomes. This process takes place in three filtering stages whose details were discussed in the ‘Merging filter pipeline’ section.

**Figure 5.**
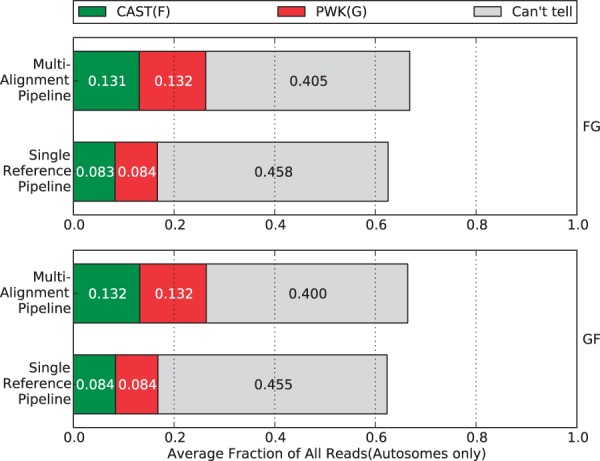
Percentage of parental origin labels in the single reference pipeline compared with the multi-alignment pipeline. The single-reference labels were generated after alignment by checking the alignment for strain-specific alleles. We removed non-autosome mappings, multi-mapped reads and reads filtered by the Random filter. In general, each strain category gains ∼5% in the multi-alignment pipeline that can be broken down into ∼2% from reads that did not map and ∼3% that were ‘Can’t tell’ in the single-reference pipeline.

We compared the performance of both pipelines in labeling read origins. To reduce bias between two crosses, we only used reads that mapped to the autosomes. The biases are introduced by mitochondrial RNA expression, which is entirely of maternal origin, and a skewing of the X-inactivation ratios in heterozygotes, which prefers genes expressed from the CAST/EiJ chromosome (21). Furthermore, reads with multiple mappings are discarded by the single-reference pipeline, which is a common strategy described by many researchers (1, 5, 10, 11). To make the comparison more fair, we ignored the mappings output by the Random filter in our pipeline. Any mappings output by the Random filter are treated as unmapped reads in the figures and tables. The percentage of reads in each category is shown in Figure 5.

Although only 4.3% more reads are processed in our method than in the traditional one, there is higher percentage of reads assigned to a unique parent of CAST or PWK. Specifically, ∼5% of reads are gained for each parental category, while the reads in the ‘can’t tell’ class are reduced by >5%.

To better understand the results, we investigated the relation between categories in the two pipelines. The results of the two reciprocal crosses are shown in Tables 3 and 4, respectively.

**Table 3.** Parental origin of reads comparison for the two pipelines from CAST × PWK samples

	Single reference pipeline				
	CAST	PWK	Cannot tell	Others	Total
Multi-alignment pipeline					
CAST	7.95%	0.12%	2.89%	2.13%	13.09%
PWK	0.12%	8.03%	2.91%	2.10%	13.16%
Cannot tell	0.03%	0.03%	39.08%	1.33%	40.47%
Others	0.18%	0.19%	0.88%	32.03%	33.28%
Total	8.28%	8.37%	45.76%	37.59%	100.00%

Note that nonautosome mappings, multi-mapped reads and Random filtered reads are in the ‘Others’ category. The diagonal represents reads labeled with the same origin in both pipelines. In all, 5.8% of reads were ‘Cannot Tell’ in the single reference pipeline but labeled as either CAST or PWK in the multi-alignment pipeline. Additionally, 4.23% were in the ‘Other’ category for the single reference, but labeled as either CAST or PWK in the multi-alignment pipeline. These two values are compared with the 0.06 and 0.37% of reads marked as CAST or PWK in the single reference pipeline, but labeled as ‘Cannot Tell’ or ‘Other’ in the multi-alignment pipeline. The net result is an increase of ∼10% for the number of reads the multi-alignment pipeline could assign a parent of origin over the single reference pipeline. Similar results for the reciprocal cross are shown in Table 4.

**Table 4.** Parental origin of reads from PWK × CAST samples

	Single reference pipeline				
	CAST	PWK	Cannot tell	Others	Total
Multi-alignment pipeline					
CAST	8.09%	0.12%	2.96%	2.05%	13.22%
PWK	0.12%	8.08%	2.95%	2.01%	13.16%
Cannot tell	0.03%	0.03%	38.73%	1.25%	40.04%
Others	0.17%	0.18%	0.87%	32.26%	33.48%
Total	8.41%	8.41%	45.51%	37.57%	100.00%

Note that nonautosome mappings, multi-mapped reads and Random filtered reads are in the ‘Others’ category.

On one hand, a large portion of reads in the CAST category of the single-reference pipeline were assigned to the same category in the multi-alignment pipeline. The percentage is around 96% for both crosses. The same percentage can be seen in the PWK category as well. This reflects that most reads with non-trivial labels in the traditional single-reference method are covered in the corresponding categories in our approach.

On the other hand, the previous 5% increase in CAST and PWK categories of our method can be attributed to the (2%) reads that cannot be aligned to the standard reference and the (3%) reads whose parental origin cannot be determined using the traditional method. This is to be expected, as our approach uses a merged set of alignments and leverages more information, such as quality score and linking, to decide the origin labels.

### Performance of merging

To evaluate the accuracy and consistency of the merging procedure, we applied the same multi-alignment pipeline to inbred samples of CAST and PWK, pretending that they are F1 hybrids crosses (i.e. we performed alignments to both pseudogenomes, annotated reads and remapped them back to the reference, and merged the results). These inbred strains can be considered as the negative controls.

**Figure 6.**
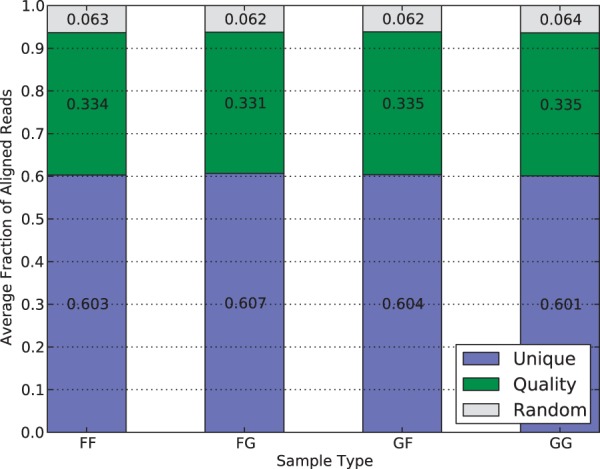
Average filter distribution of mapped reads for diallel samples in the multi-alignment pipeline. F and G denote CAST and PWK, respectively. As each category is approximately equal, it suggests that there is no inherent bias to a strain caused by the filters.

Figure 6 shows the percentage of mapped reads that are outputted by each of the three filters. Around 60% of reads are merged in the Unique filter step, suggesting they have either unique mappings in one of the pseudogenomes or identical mappings in both of them. Another 33% reads have multiple pseudogenome mappings with one mapping better than the rest, so they are filtered by quality score. The remaining 6% have multiple mappings with identical quality scores, so one was randomly chosen to be reported in the final output. The consistency of filtering percentages in different strains, including the hybrid crosses and inbreds, suggests that the filters in the merging process do not bias the result.

**Figure 7.**
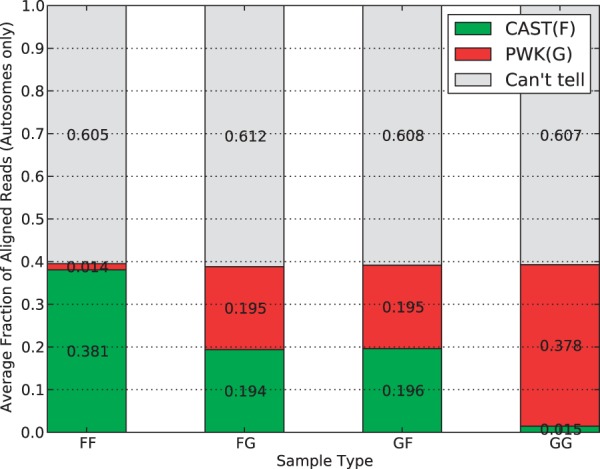
Average parent-of-origin distribution of autosome mapped reads for multiple sample types in the multi-alignment pipeline. In all sample types, ∼60% are ‘can’t tell’ with the remaining 40% divided into parent categories. For inbreds, the vast majority (38%) are from the associated parent category with only 2% having a better mapping to the other pseudogenome. For the F1 hybrids, the categories are roughly equal, which is expected for the autosomes.

In Figure 7, we show the percentage of mapped reads in each of the parental origin categories. To avoid the bias caused by the X chromosome and mitochondria, only reads mapping to autosomes were considered in this analysis. Around 60% of reads fall into the ‘Can’t tell’ class, and this percentage is consistent in F1 hybrids (the second and the third) as well as negative controls (the first and the last). For the residual 40%, we can see the ratio of CAST reads to PWK reads is 1:1 for the F1 hybrids, which is expected because reads are equally likely to come from either parent in autosomes. The ratio for inbred strains, however, is different. In fact, the majority of the 40% are classified to the corresponding inbred strain, whereas only 1.4–1.5% are mislabeled. This error rate is likely caused by sequencing noise or unannotated parental alleles.

## Discussion and Conclusion

Although we only applied our pipeline to RNA-seq short reads in this experiment, it is also applicable to other quantitative high-throughput sequence analysis tasks, such as DNA-seq, Chip-seq, DNase-seq, Bis-seq, etc. For example, studies of allele-specific copy number variations can leverage our pipeline for DNA-seq data. The resulting read-origin annotations can be used to estimate the number of DNA copies in different parental haplotypes in later analysis steps.

Although we chose to use a diallel experiment to evaluate our new pipeline in the ‘methods and result’ sections, it is equally applicable to other multi-parental crosses. For example, our multi-alignment pipeline can be directly applied to recombinant inbred lines (RILs) [22] and backcrosses. For a multi-parental cross with *N* distinct inbred founders, we would generate *N* pseudogenomes and perform *N* separate alignments. These alignments can then be merged using *N* BAM files. In this scenario, each mapping that is saved to the output will have an *N*-bit flag set indicating which files the read was found in. This allows for cases where a mapping’s origin is shared/ambiguous between multiple founders. The latest version of Suspenders allows for a variable number of input alignments during the merging process.

Furthermore, we can incorporate additional filters into the pipeline to better determine the origin of mappings. In our experiment, we only used the Unique and Quality filters as informative filters. This resulted in ∼5% of the mapped reads being handled by the Random filter. Adding an additional filter before the Random filter will help to reduce the amount of random choices made in the final output. One possible filter is a Pileup filter based on choosing among otherwise equal mappings the single mapping that has the most surrounding mappings supporting it. To do this, we first find all mapping sets that can be filtered by the Unique or Quality filters and use their chosen mappings to compute the read coverage at each base in the reference genome. Then, any mapping sets that could not be resolved using Unique or Quality would compare the pileup coverage of each potential mapping in the set and choose the mapping with the highest coverage. This will be particularly useful for reducing the number of reads that map to pseudogenes in RNA-seq. In cases where the pileups are not significantly different, more computation or simply using the Random filter may be necessary. Suspenders currently has a preliminary version of this filter included in the software package.

To summarize, we propose a new multi-alignment pipeline, which is generic enough to handle reads of various types of organisms from different high-throughput sequencing techniques. We demonstrated its effectiveness on RNA-seq data from a diallel cross and compared our pipeline with a single-reference pipeline. It is shown that our pipeline outperforms the traditional single-reference-based alignment approaches: not only are more reads aligned by our pipeline, but a higher percentage of them are assigned a correct origin.

The two key components of our pipeline, Lapels and Suspenders, are Python scripts that can be downloaded at https://code.google.com/p/lapels/ and https://code.google.com/p/suspenders/.
